# Draft Genome Sequences of Two Clinical Isolates of *Mycobacterium tuberculosis* from Sputum of Kazakh Patients

**DOI:** 10.1128/genomeA.00466-15

**Published:** 2015-05-14

**Authors:** Ulykbek Kairov, Ulan Kozhamkulov, Askhat Molkenov, Saule Rakhimova, Ayken Askapuli, Maxat Zhabagin, Ainur Akhmetova, Dauren Yerezhepov, Zhannur Abilova, Aliya Abilmazhinova, Venera Bismilda, Leila Chingisova, Zhaxybay Zhumadilov, Ainur Akilzhanova

**Affiliations:** a Department of Genomic and Personalized Medicine, Center for Life Sciences, Nazarbayev University, Astana, Kazakhstan; b National Center of Tuberculosis Problems of the Republic of Kazakhstan, Almaty, Kazakhstan

## Abstract

Here, we report the draft genome sequences of two clinical isolates of *Mycobacterium tuberculosis* (MTB-476 and MTB-489) isolated from sputum of Kazakh patients.

## GENOME ANNOUNCEMENT

Tuberculosis remains one of the major problems in public health, with 9 million new cases and the deaths of around 1.5 million people each year (1). During the last 10 years the tuberculosis incidence and mortality rate in Kazakhstan decreased to 52.4% and 72.8%, respectively, but high tuberculosis incidences and mortality rates still exist. Thus, the tuberculosis incidence and mortality rate were 73.4 and 5.6 per 100,000 people in 2013, respectively, whereas in 2012 the same indicators were 81.7 and 7.4 per 100,000 (2).

Despite progress in decreasing the global incidence of drug-susceptible tuberculosis, the existence of multidrug-resistant (MDR) and extensively drug-resistant (XDR) tuberculosis has led to decreasing efficiency of chemotherapy and prolonged treatment (1). Kazakhstan is 1 of 18 countries in the WHO European Region with a high rate of MDR tuberculosis (2).

It is extremely important to examine sensitive and resistant strains with different mutations in genes encoding drug metabolism among *Mycobacterium tuberculosis* strains from the different geographic regions of Kazakhstan. Some previous studies have been performed in Kazakhstan by using Sanger sequencing and genotyping methods (3, 4). Here, we report the draft genome sequences of two clinical isolates of *M. tuberculosis*, isolated from sputum of Kazakh patients, performed by using a next-generation sequencing platform.

Material collection, sputum and DNA extraction, and determination of drug sensitivity of two *M. tuberculosis* isolates MTB-476 and MTB-489 were performed at the reference laboratory “National Center of Tuberculosis Problems,” Kazakhstan. DNA shotgun libraries were prepared by using a GS FLX Titanium rapid library preparation kit. Whole-genome sequencing of the two *M. tuberculosis* isolates was performed by using a Roche 454 GS FLX+ next-generation sequencing (NGS) platform at the Centre for Life Sciences, Nazarbayev University, Kazakhstan.

The run yielded 96 Mb and 104.2 Mb, with 184,296 and 176,838 passed filters, average lengths of 520.2 and 589.3 bp, and 21.8× and 23.7× coverage for the MTB-476 and MTB-489, respectively. The sequencing reads were then *de novo* assembled into contigs using Newbler v.2.8. The draft genome annotations were performed using the NCBI Prokaryotic Genome Annotation Pipeline (http://www.ncbi.nlm.nih.gov/genomes/static/Pipeline.html) and xBASE, which uses Glimmer (5) for gene prediction, and tRNAsacn-SE (6) and RNAmmer (7) for RNA prediction. The contigs were aligned and ordered against *M. tuberculosis* H37Rv using Mauve v.2.3.1 (8). Synteny evaluation and visualization were carried out using C-Sibelia (9) and Circos (10). The genome of *M. tuberculosis* MTB-476 consists of 257 contigs with a G+C content of 65.2% and 4,204 predicted coding sequences, 46 tRNAs, and 3 rRNAs. The genome of *M. tuberculosis* MTB-489 has 187 contigs, with G+C content of 65.3% and 4,183 predicted coding sequences, 45 tRNAs, and 3rRNAs. This draft genome data may provide a basis for creation of the reference database, the subsequent study, and comparison with the different drug-resistant *M. tuberculosis* isolates circulating in Kazakhstan.

### Nucleotide sequence accession numbers. 

The draft genome sequences of the *M. tuberculosis* isolates MTB-476 and MTB-489 have been deposited at DDBJ/EMBL/GenBank under the accession numbers AZBA00000000 and AZAZ00000000. The versions described in this paper are the first versions, accession numbers AZBA01000000 and AZAZ01000000 (BioProject PRJNA229943 and PRJNA229941).
